# Proximal Tibia Primary Leiomyosarcoma: A Case Report and Review of Literature

**DOI:** 10.7759/cureus.43712

**Published:** 2023-08-18

**Authors:** Sandeep Kumar Yadav, Aakash Choudhary, Prabodh Kantiwal, Meenakshi Rao, Abhay Elhence

**Affiliations:** 1 Orthopaedics, All India Institute of Medical Sciences, Jodhpur, IND; 2 Pathology, All India Institute of Medical Sciences, Jodhpur, IND

**Keywords:** actin, desmin, h-caldesmon, multi-speciality, megaprosthesis, smooth muscle differentiation, multidisciplinary, leiomyosarcoma, chemotherapy, bone

## Abstract

The paper at hand presents a unique case of leiomyosarcoma (LMS) involving the left leg in a 56-year-old patient. This individual experienced pain and the presence of a mass for approximately eight months before seeking medical attention. A diagnostic biopsy revealed the presence of multinucleated pleomorphic cells arranged in intersecting fascicles upon immunohistochemistry (IHC) staining for vimentin, caldesmon, and smooth muscle actin. The rarity of LMS in the extremities highlights the need for further understanding and research to determine the most suitable treatment approaches for such patients. In this specific case, the patient underwent tumor excision followed by reconstruction using a megaprosthesis. This report emphasizes the importance of considering unique treatment strategies when dealing with rare neoplasms like LMS in the extremities. As medical knowledge continues to evolve, gaining insights into the optimal management of such cases will be crucial for improving patient outcomes and overall prognosis.

## Introduction

Leiomyosarcoma (LMS) is a prevalent type of soft tissue sarcoma (STS), constituting approximately 7% to 10% of all STS cases [1]. On the other hand, LMS of the bone is an infrequent occurrence, accounting for less than 0.7% of cases, and tends to develop in uncommon locations [2]. The retroperitoneum and the genitourinary tract are the two anatomical regions where these tumours most frequently appear. There is an equal percentage of LMS prevalence across all age groups [3].

Its characteristic appearance on plain radiographs is that of a lytic lesion without a surrounding sclerotic rim. Immunohistochemistry (IHC) plays a crucial role in diagnosis, with positive staining for smooth muscle actin (SMA), desmin, and h-caldesmon often observed [4].

Given the complexity of LMS cases, successful management requires a multidisciplinary approach involving various medical specialties. However, the cornerstone of treatment remains complete surgical resection of the tumour. Following surgery, additional therapeutic measures such as chemotherapy and radiotherapy are utilized to provide complementary support.

This report highlights a distinctive case of LMS originating from the proximal tibia. The tumour was successfully treated through a comprehensive approach involving a complete, wide resection of the affected area. Additionally, joint reconstruction using a megaprosthesis was employed to restore the function of the affected bone.

## Case presentation

**Figure 1:**
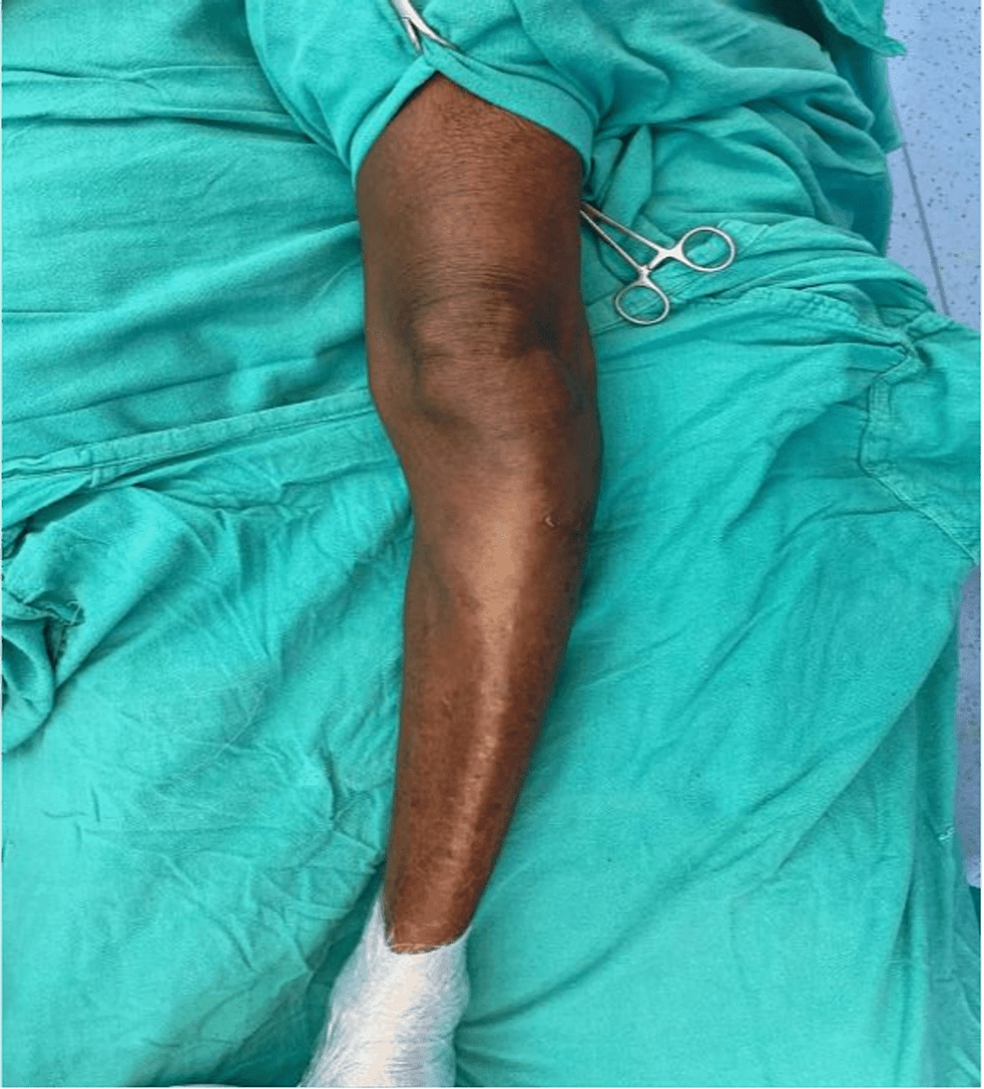
Left leg showing swelling around the proximal aspect

**Figure 2:**
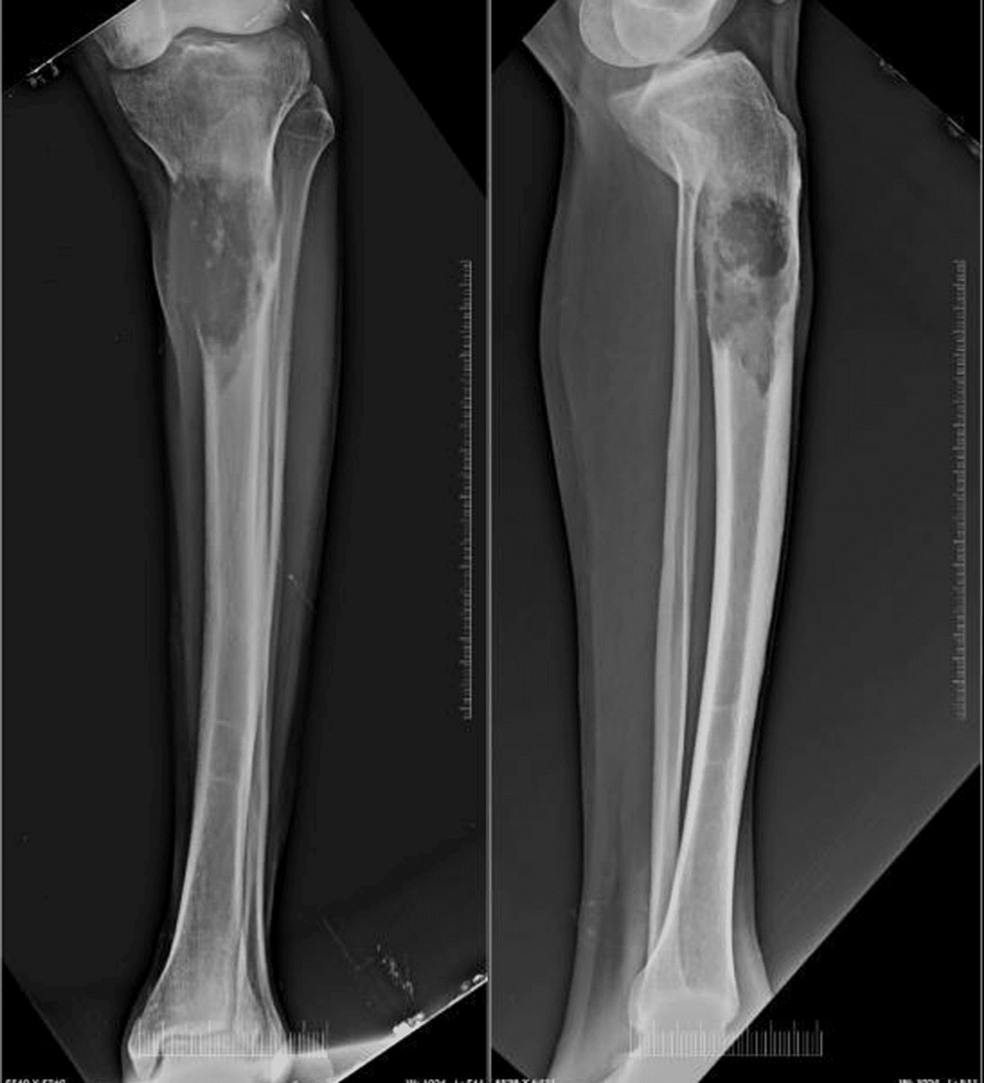
Preoperative radiograph showing lytic lesion in the metaphysio-diaphyseal region A: Anteroposterior view of the leg, B: Lateral view of the leg

A 56-year-old male presented to our outpatient department (OPD) with the chief complaint of pain and swelling in his left knee for eight months (Figure 1). The pain had aggravated over the last three months. His past history did not include any significant comorbidity or past surgery. Local examination revealed a bony, hard swelling over the left proximal leg region measuring approximately 5x5x7 cm. There was no distal neurovascular deficit. His radiographs showed the presence of a lytic lesion in the metaphysio-diaphyseal region of the left proximal tibia (Figure 2).

**Figure 3:**
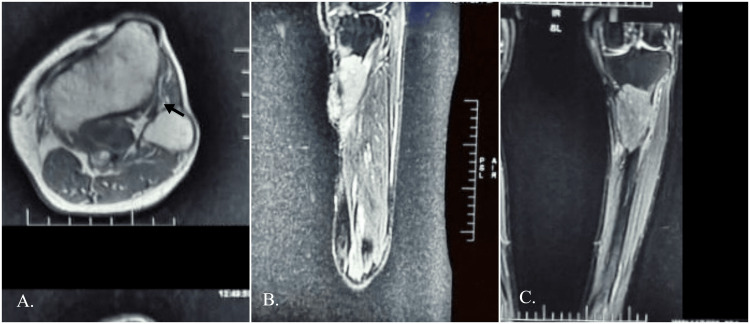
MRI sections showing lytic lesion in the proximal tibia A: Axial view shows the tumour confined to the tibia with an intact interosseous membrane (black arrow); B: Sagittal view, C: Coronal view

An MRI of the left leg revealed a heterogenous expansile space-occupying lesion (SOL) in the upper 1/3rd shaft of the tibia, destroying the bone and breaking the continuity of the cortex. It was hypointense in T1-weighted images while hyperintense in T2-weighted and fat-suppressed inversion recovery images. Extraosseous extension of SOL was seen outside the cortex, involving muscles and extending even up to the subcutaneous fatty tissue. The lesion measured 49x52x80 mm (anteroposterior (ap) x transverse (tr) x superior to inferior (si)). There was altered bone marrow signal intensity suggestive of bone marrow edema infiltration in the tibia around SOL, while normal bone marrow signal intensity was seen in the fibula. The major neurovascular bundle appeared normal (Figure 3). 

These findings were suggestive of either chondrosarcoma, metastasis, or even myeloma. To rule out differentials, a renal function test was performed, which revealed normal blood creatinine and serum calcium with negative urine Bence-Jones protein. A PET-CT was performed, and metastatic lesions were found in the brain (right frontal region), lungs (subpleural nodules in both lungs), inguinal region, right femur, and right iliac bone.

**Figure 4:**
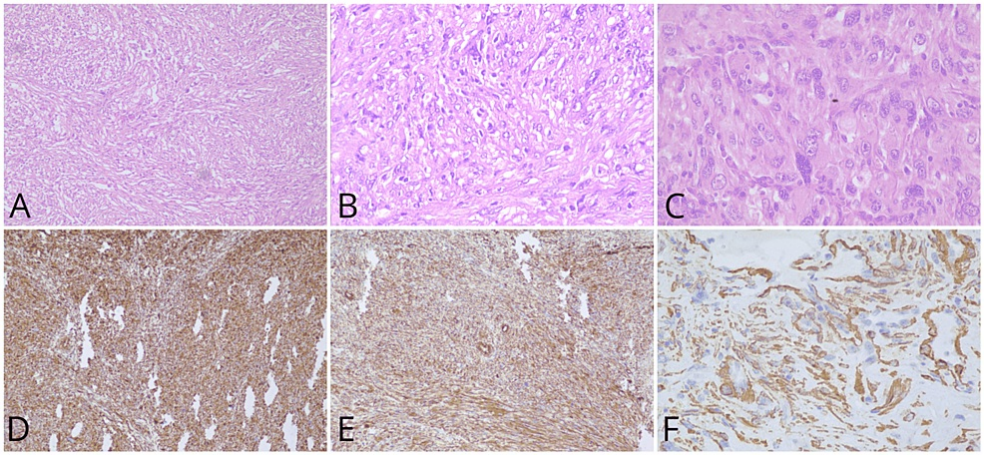
Biopsy findings A: Low power view showing a spindle cell tumour arranged in intersecting fascicles, H&E stain, 10x; B: High power view showing mildly pleomorphic tumour cells, with eosinophilic to vacuolated cytoplasm, H&E stain, 40x; C: High power view showing tumor cells exhibiting increased pleomorphism with occasional multinucleated tumor cells, H&E stain, 40x; D: The tumor cells are positive for vimentin on IHC, 10x; E: The tumor cells are positive for caldesmon on IHC, 10x; F: The tumor cells are positive for SMA on IHC, 40x H&E: Hematoxylin and eosin, IHC: Immunohistochemistry, SMA: Smooth muscle actin

The patient underwent a core needle biopsy from the left proximal tibia and was reported to have fibrocollagenous tissue with an invasive tumour arranged in fascicles and whorls with nuclear pleomorphism and eosinophilic cytoplasm. It was positive for vimentin, CK, S100, and SMA on IHC (Figure 4). These findings were consistent with LMS, as it shows deeply eosinophilic palisade-arranged cells with S100, vimentin, and SMA-positive IHC. 

**Figure 5:**
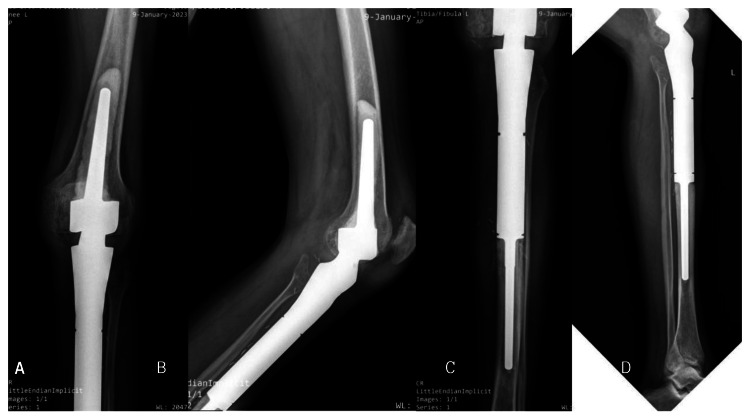
Postoperative radiograph of the left knee with a megaprosthesis in-situ A: Anteroposterior view of the knee, B: Lateral view of the knee, C: Anteroposterior view of the leg, D: Lateral view of the leg

After a complete evaluation, the patient underwent a wide resection and reconstruction with a megaprosthesis and medial gastrocnemius flap (Figure 5).

The patient withstood the surgery well. There were no postoperative complications. He was later admitted to the medical oncology department for postsurgical excision of the LMS proximal tibia in view of adjuvant chemotherapy with an ifosfamide-adriamycin regimen and is under regular follow-up in our department. Currently, after five months of surgery, the patient is well, with no evidence of any recurrence or metastasis with ongoing follow-up.

## Discussion

Leiomyosarcoma is a spindle-cell sarcoma variation and a kind of STS. They account for 7% to 10% of soft tissue sarcomas overall [1]. Skin, gastrointestinal, uterine, and somatic lesions are other subcategories of LMS [2,3]. Less than 0.7% of all primary malignant bone tumours occur as primary LMS of the bone, which is relatively low in occurrence [2]. Bones are also susceptible to LMS, especially long bones in the lower extremity involving the femur or tibia's metaphyseal region [4,5]. Even though visceral metastases are more frequent, bone metastases are not uncommon and can cause serious morbidities in the patient [6].

There has been an equal incidence of LMS reported in men and women. Age predilection has been the subject of inconsistent findings. One set of authors claimed that this tumour was more prevalent in people in their seventh and eighth decades of life, while other researchers claimed that there was an equal distribution of ages [3,7].

There are actually two competing hypotheses for the origin of primary bone LMS. The first postulate demonstrates the involvement of vascular smooth muscle cells in the bones. The second postulate maintains that intermediate cells, in particular fibroblasts, can differentiate into smooth muscle cells that are capable of producing a matrix of connective tissue and myofilaments [5,8,9].

The most common signs are discomfort and localized swelling. Large tumours can be palpated as they spread beyond the bony structures. It has been documented that 15% of patients exhibit pathological fractures as a result of the osteolytic nature of the lesion causing cortical damage [10].

Leiomyosarcoma of the bone presents as a lytic lesion with fuzzy borders and a permeative pattern on plain radiographs. Bone lesions exhibit osteolysis as well as varying degrees of assertiveness, such as the absence of a sclerotic rim and cortical breach [3,11,12]. Bone LMS often exhibits hypointense signal strength on T1-weighted images and heterogeneous signal intensity on T2-weighted images [13]. In our patient, MRI showed similar pictures of a hypointense T1-weighted image and a hyperintense T2-weighted image, consistent with those described in the literature for LMS.

The LMS in the bone has similar histopathological characteristics as other sites, showing elongated cells with abundant cytoplasm, sporadic vacuoles, and pleomorphic nuclei. It is eosinophilic, and the fascicles of these cells permeate the stroma. In high-grade tumours, there is evidence of mitotic activity, including aberrant forms [14]. The most important aspect of smooth muscle differentiation connected to an LMS diagnosis is that LMS immunohistochemically has palisade-arranged cells with eosinophilic cytoplasm and expresses actin, desmin, S100, vimentin, SMA, and h-caldesmon [15]. A marker that appears to be more specific is h-caldesmon [16]. In our patient, we reported having fibrocollagenous tissue with an invasive tumour arranged in fascicles and whorls with nuclear pleomorphism and deeply eosinophilic cytoplasm. It was positive for vimentin, CK, S100, and SMA on immunohistochemistry, and these findings were consistent with LMS.

Prior to surgery, imaging examinations must be used to determine the degree and extent of the surgical resection. A thoracic-abdomen-pelvic CT scan and MRI are advisable to determine the ideal spread of the tumour and any occurrence of skip-metastasis. A PET-CT may help with staging, grading, and assessing the effectiveness of neoadjuvant therapy [14].

The most effective form of treatment and the only curative one is surgical resection. The R0 resection provides better outcomes microscopically than the R1 resection; therefore, the resection must be large with free margins [17].

Another therapeutic option is radiotherapy; however, its effectiveness is debatable. Antonescu et al. found similar survival among patients who underwent surgery alone and those who underwent radiation therapy following surgery, possibly because this form of tumour is resistant to radiation [18].

The primary strategy for metastatic LMS is chemotherapy. The most effective medications for treating bone sarcoma include cisplatin, doxorubicin or doxorubicin-based chemotherapy, and dacarbazine, which are typically used in first-line metastatic situations [19].

The evolution and survival rate appear to be unaffected by anatomical location and histological grade. A retrospective trial by Antonescu et al. showed hardly any difference in life span between patients who got chemotherapy and those who received surgery [20]. Age ≥40, size greater than 8 cm, the existence of a pathological fracture, limb disarticulation, R1 margins microscopically, and an inadequate response to preoperative treatment were the most significant prognostic variables linked with a reduction in survival [20]. The prognosis was satisfactory for our patient, who was over 40 years old and had a lesion with a maximal diameter of 8 cm but no pathological fracture and an R0 margin resection after surgery.

Our patient with metastatic bony LMS was treated with wide resection and reconstruction with a megaprosthesis right lower limb and medial gastrocnemius flap following multidisciplinary evaluation. He is currently leading a healthy life, with no evidence of local recurrence reported on follow-ups. He continues to be followed up at our institute's medical oncology department for adjuvant chemotherapy with the ifosfamide-adriamycin regimen.

## Conclusions

In this study, we presented a case of LMS occurring in the proximal tibia of a 56-year-old male, along with metastasis. To gain a better understanding of this case, we conducted a comprehensive evaluation, comparing it with other similar cases documented in the literature. The patient’s response to treatment was highly positive, as he showed significant improvement after undergoing wide resection and megaprosthesis. Notably, he experienced relief from pain and showed no signs of local recurrence during the follow-up period. When a patient presents with swelling in or around the knee region, it is essential to undertake a multidisciplinary evaluation. This process should involve various diagnostic methods, such as imaging, histological examination, and IHC studies. Considering the potential spectrum of differential diagnoses, it is crucial to include LMS as one of the possible considerations. This case highlights the importance of thorough evaluation and diagnosis when dealing with knee swelling, as prompt and accurate identification of LMS can lead to timely and appropriate treatment strategies. The success of wide resection and megaprosthesis in this case underscores the significance of tailored approaches in managing LMS, offering potential positive outcomes for patients with similar conditions. Continuous research and analysis of such cases in the medical literature contribute to advancing our understanding and refining treatment protocols for this rare but impactful neoplasm.
